# The impact of drug shortages on drug prices: evidence from China

**DOI:** 10.3389/fpubh.2023.1185356

**Published:** 2023-11-08

**Authors:** Shuchen Hu, Jinwei Zhang, Jianwei Li, Jieqiong Zhang, Mengyuan Pan, Cheng Xiang, Chintan V. Dave, Caijun Yang, Yu Fang

**Affiliations:** ^1^Department of Pharmacy Administration and Clinical Pharmacy, School of Pharmacy, Xi’an Jiaotong University, Xi'an, China; ^2^Center for Drug Safety and Policy Research, Xi’an Jiaotong University, Xi'an, China; ^3^School of Software Engineering, Xi’an Jiaotong University, Xi'an, China; ^4^Center for Pharmacoepidemiology and Treatment Science, Institute for Health, Health Care Policy and Aging Research, Rutgers University, New Brunswick, NJ, United States; ^5^Department of Pharmacy Practice and Administration, Ernest Mario School of Pharmacy, Rutgers University, Piscataway, NJ, United States

**Keywords:** drug shortage, drug price, mixed-effects model, drug policy, subgroup analysis

## Abstract

**Introduction:**

Drug shortages pose a serious global public health challenge, affecting China and other countries. Evidence from USA shows that short-supplied drugs demonstrated a very high price growth during and after a shortage. However, the effect of shortages on drug prices in China remains unknown. This paper aims to understand the impact of drug shortages on prices and explore implications for shortage prevention policy.

**Methods:**

We collected the purchase prices and delivery rates of 120 drugs from April 2019 to December 2021 across whole China. We examined price progression of affected drugs using linear mixed-effects models and performed subgroup analyses based on the number of manufacturers and the severity of shortage.

**Results:**

Non-shortage cohort had an annual price growth of 11.62% (95% confidence interval [CI] 8.34 to 14.98). Shortage cohort demonstrated an annual price growth of 8.08% (95%CI 0.12 to 16.77) in the period preceding a shortage, 27.57% (95%CI 6.17 to 52.87) during a shortage, and 9.38% (95%CI −12.64 to 36.39) in the post-shortage period. Drug shortages’ impact on prices varied across subgroups. Compared with that of drug markets supplied by a single manufacturer, the price growth rate of markets supplied by more than one manufacture declined more after the shortage resolution.

**Conclusion:**

Shortages resulted in significant price increases of study markets, especially the low-priced markets, while the shortage resolution slowed the growth. The primary shortage driver has shifted from the low price to others drivers, such as unavailability of active pharmaceutical ingredients. For currently sole-supplied drugs, the expedited review of applications from other manufacturers should be considered.

## 1. Introduction

Drug shortages pose a serious global public health challenge (1), affecting China (2–5) and other countries (6–8). During the last decade, clinically necessary agents, such as protamine, (9) methimazole (10), norepinephrine (11), mercaptopurine (12) and nitroglycerin (13), have been reported to be in short supply across China. From January to May 2018, the Chinese National Drug Shortage Monitoring Platform reported shortages of 1947 drugs (14). Such shortages can disrupt patient care – resulting in worse health outcomes. When drug prices rise subsequent to a shortage, patients and hospitals are faced with the dual problems of reduced drug availability and a more expensive remaining drug supply (15–21).

Although the factors contributing to the phenomenon of drug shortages are likely multi-modal, low drug prices – and consequently, low drug profits, have been identified as a key contributor of drug shortages (22–24). Historically, drug prices in China were kept low through a provincial-level drug tender bidding system (25). Briefly, for each province, a bidding system was used to select the drug manufacturers that would supply the medication over a set price point and duration. Accordingly, manufacturers were incentivized to bid artificially lower prices that were not allowed to be altered for the duration of the contract, regardless of external factors such as rise in prices for input of drugs or distribution costs (26). As a result, drug shortages were common as manufacturers prioritized the supply of more profitable products over less profitable ones (22–24, 26).

To address this issue, the central government introduced several policies, including the 2014 Low-Price Drug Policy – raising the price cap of lower priced drugs (27), the 2015 pharmaceutical price reform – deregulating the price of drugs (28), and the 2016 “Zhi Jie Gua Wang” procurement policy – abolishing the bidding process entirely for a select basket of critical medications (e.g., pediatric, gynecological, and emergency drugs) (25). While some evidence has shown that these policies resulted in a corresponding increase in drug prices in the ensuing years (29–31), drug shortages continue to pose a significant challenge to the Chinese healthcare system (13, 14).

Evidence from the USA shows that shortages usually affect medications of limited profitability, and lead to persistent price increases. This suggested that low profitability was the primary shortage driver in the USA. To our knowledge, no prior study has examined the impact of shortages on drug prices on a national scale and whether the low price was still the primary driver in China. The utility of prior studies examining this topic are limited by several factors including their qualitative designs (32–36), or use of data derived from a single region within China (37–39). Moreover, given the unique characteristics of the Chinese healthcare system, it is unclear whether similar data from the US or other countries are generalizable to China (15, 19, 40–42). Accordingly, we sought to examine the impact of drug shortages on price fluctuations using a national Chinese database to identify the primary driver in China and explore the implication for shortage prevention policy.

## 2. Methods

### 2.1. Data source and study cohort

We extracted monthly procurement data from April 2019 to December 2021 from 31 provincial Centralized Drug Procurement Service Centers. The data are inclusive of all public medical institutions (538,767 institutions) in China, and provide information on the drug name, dosage form, strength, quantity ordered, quantity delivered, total expenditure, and drug manufacturer. We focused on the hospital sector in this research, as patients acquire most of their medications from hospitals in China.

In China, a shortage is defined when an approved drug with characters of clinical necessity and irreplaceability, is in short or unstable supply during a period in an area (43). Based on this definition, the National Health Commission released two lists in December 2020: the National Shortage Drug List, which includes drug products that experienced a shortage in more than three provinces; and the National Key Monitoring List, which reflect drugs that are at high risk of experiencing a national shortage (44). The two lists included 57 chemical entities totally, corresponding to 163 unique drug products representing distinct combinations of active ingredient, dosage, and administration form (44). As drug prices and shortage status vary according to province, we considered each unique drug-province pair as an individual drug market (e.g., cytarabine for injection (0.1 g) in Beijing). This study follows the Strengthening the Reporting of Observational Studies in Epidemiology (STROBE) reporting guideline (45).

### 2.2. Evaluation indicators

The study period was segmented into 33 monthly intervals, and the unit price of each drug market was calculated by dividing the overall drug expenditure for that product by the quantity of the drug purchased. All prices were adjusted to 2019 levels using the consumer price index (46). Drug shortages were quantified by measuring the monthly delivery rate, which was calculated by dividing the actual delivery quantity by the total quantity of product ordered (47–50). A drug market was considered to be experiencing a shortage if the calculated monthly delivery rate was less than 40%, as recommended by the National Healthcare Security Administration. Despite the Chinese National Health Commission requires healthcare workers to report drug shortages occurring in public medical institutions through the drug shortage reporting platform, real-time shortage information on the platform is not publicly accessible and was not considered in the research.

For each drug market, the study period was classified into the following three mutually exclusive categories: (i) the pre-shortage period, which was comprised of months prior to a drug shortage; (ii) the shortage period, which was comprised of months that the drug was experiencing a shortage; and (iii) the post-shortage period, which represented the months following the resolution of a drug shortage. As a drug market may have experienced more than one shortage, we only examined the longest-lasting shortage. If a drug market had more than one shortage with the same duration, the first one was selected.

To better reflect disruptions in drug supply and prices following the resolution of a shortage, we considered the 5 months subsequent to the shortage as the shortage period. We also conducted sensitivity analyses across alternative scenarios where we included the following two, three, four, or all months after the resolution of the drug shortage as the shortage period. For intermittent shortages lasting only 1 month, we anticipated the residual impact on prices to be minimal, and thus only considered the following 2 months as the shortage period.

### 2.3. Statistical analysis

We collected 120,271 procurement observations for the 163 drugs across the 31 provinces. As not all 31 provinces had access to the 163 products, this resulted in an initial cohort of 2,713 drug markets. We further restricted the study cohort to the 2097 drug markets (belonging to 120 drugs) that had at least 11 months of available data (Supplementary Appendix 1).

We conducted three separate analyses, each adopting a mixed-effects linear regression model. All models controlled for the fixed effects of shortage status, drug class, and the number of manufacturers, and employed random intercept terms for each drug market and random slopes for the effect of time.

To examine whether pre-shortage prices for drugs that experienced a shortage differed from their counterparts that did not experience a shortage, the first analysis examined the differences in monthly price growth in markets with and without a shortage. More specifically, pre-shortage prices for shortage markets and all available prices for non-shortage markets were included in this analysis. In the second analysis, we examined the difference in monthly price growth for markets that experience a drug shortage by comparing their pre- and during-shortage prices. Accordingly, data from the post-shortage period was excluded for this analysis. Finally, in order to examine changes in the price during and after the resolution of a shortage, we compared prices during- and post-shortage, excluding pre-shortage data for analysis. All the model equations and additional descriptions are outlined in Supplementary Appendix 2.

For the second and third analyses, we performed two subgroup analyses based on whether the drug market was supplied by a sole manufacturer or not, as well as the severity of the shortage (median delivery rate: 24.15%), respectively (Supplementary Appendix 3). We used R version 4.2.0 (RStudio) for all sections of analysis.

Lastly, based on the first and last month prices of each market, we calculated the price growth for each market. Using information on shortage status (i.e., if a drug market experienced a shortage anytime during the study period) and the observed empirical baseline median price (11.5 RMB, about 1.7$), we divided all the markets into four subgroups namely: non-shortage and low-priced subgroup (*N* = 451), non-shortage and high-priced subgroup (*N* = 469), shortage and low-priced subgroup (*N* = 601) and shortage and high-priced subgroup (*N* = 576). Thereafter we compared the price growth rate for two cohorts and four subgroups, respectively.

## 3. Results

We included 2097 markets, of which 1,177 (56.1%) were affected by a shortage during the study period. The top three affected drug classes were cardiovascular system agents (20.0%), antineoplastic and immunomodulating agents (17.0%), and blood & blood forming agents (14.8%) (Table 1). During the study period, the monthly proportion of markets experiencing a shortage decreased, and on average 11.1% (95%CI 10.2 to 12.0) of the markets experienced a shortage in any given month (Supplementary Appendix 4).

**Table 1:** Characteristics of studied markets.

	Affected by shortage (*n* = 1,177)	Not affected by shortage (*n* = 920)	Total (*n* = 2,097)
Median shortage initiation month (IQR)	March 2020 (September 2019 to November 2020)	–	
Medication class, n (%)			
Cardiovascular system	235 (20.0)	234 (25.4)	469 (22.4)
Antineoplastic and immunomodulating agents	200 (17.0)	119 (12.9)	319 (15.2)
Blood and blood forming organs	174 (14.8)	145 (15.8)	319 (15.2)
Systemic hormonal preparations, excl. sex hormones and insulins	129 (11.0)	127 (13.8)	256 (12.2)
Alimentary tract and metabolism	74 (6.3)	53 (5.8)	127 (6.1)
Nervous system	65 (5.5)	39 (4.2)	104 (5.0)
Respiratory system	47 (4.0)	36 (3.9)	83 (4.0)
Musculo-skeletal system	45 (3.8)	20 (2.2)	65 (3.1)
Antiinfectives for systemic use	24 (2.0)	21 (2.3)	45 (2.1)
Various	184 (15.6)	126 (13.7)	310 (14.8)

The study markets are drug-province pairs, such as cytarabine for injection (0.1 g) in Beijing (one market). The markets ever experiencing a shortage were included in the shortage cohort and the others were the non-shortage cohort.

### 3.1. Analysis 1: comparison of price growth in pre-shortage periods to markets that did not experience a shortage

We compared the logarithmic pre-shortage price growth in 926 markets that experienced a shortage (251 markets were excluded due to lack of pre-shortage data) compared to 920 drug markets that did not experience a shortage. The slope observed was 0.0065 and 0.0092 for the pre-shortage and non-shortage cohort respectively, corresponding to a non-significant difference in monthly price growth of (−0.0026, 95%CI −0.0066 to 0.0013; *p* = 0.19) (Table 2).

**Table 2:** Logarithmic price trends in pre-shortage, shortage and post-shortage periods.

Main analysis	Value (95%CI)	*P* value
Comparison of price growth in non-shortage periods		
Slope		
Non-shortage cohort	0.92% (0.67, 1.17%)	<0.001
Shortage cohort	0.65% (0.01, 1.30%)	
Difference	−0.26% (−0.66, 0.13%)	0.19
Other fixed effects		
Medication class	6.89% (4.62, 9.16%)	<0.001
Manufacturers	−14.03% (−17.61, −10.46%)	<0.001
Comparison of pre-shortage and shortage periods		
Slope		
Pre-shortage	0.36% (0.03, 0.69%)	0.03
During shortage	2.05% (0.50, 3.60%)	
Difference	1.69% (0.47, 2.92%)	0.007
Other fixed effects		
Medication class	10.95% (7.47, 14.44%)	<0.001
Manufacturers	−13.64% (−21.22, −6.05%)	<0.001
Comparison of shortage and post-shortage periods		
Slope		
During shortage	3.76% (2.91, 4.62%)	<0.001
Post-shortage	0.75% (−1.12, 2.62%)	
Difference	−3.01% (−4.03, −2.00%)	<0.001
Other fixed effects		
Medication class	10.80% (7.44, 14.16%)	<0.001
Manufacturers	−18.85% (−25.76, −11.94%)	<0.001

### 3.2. Analysis 2: comparison of pre-shortage and shortage periods

**Figure 1:**
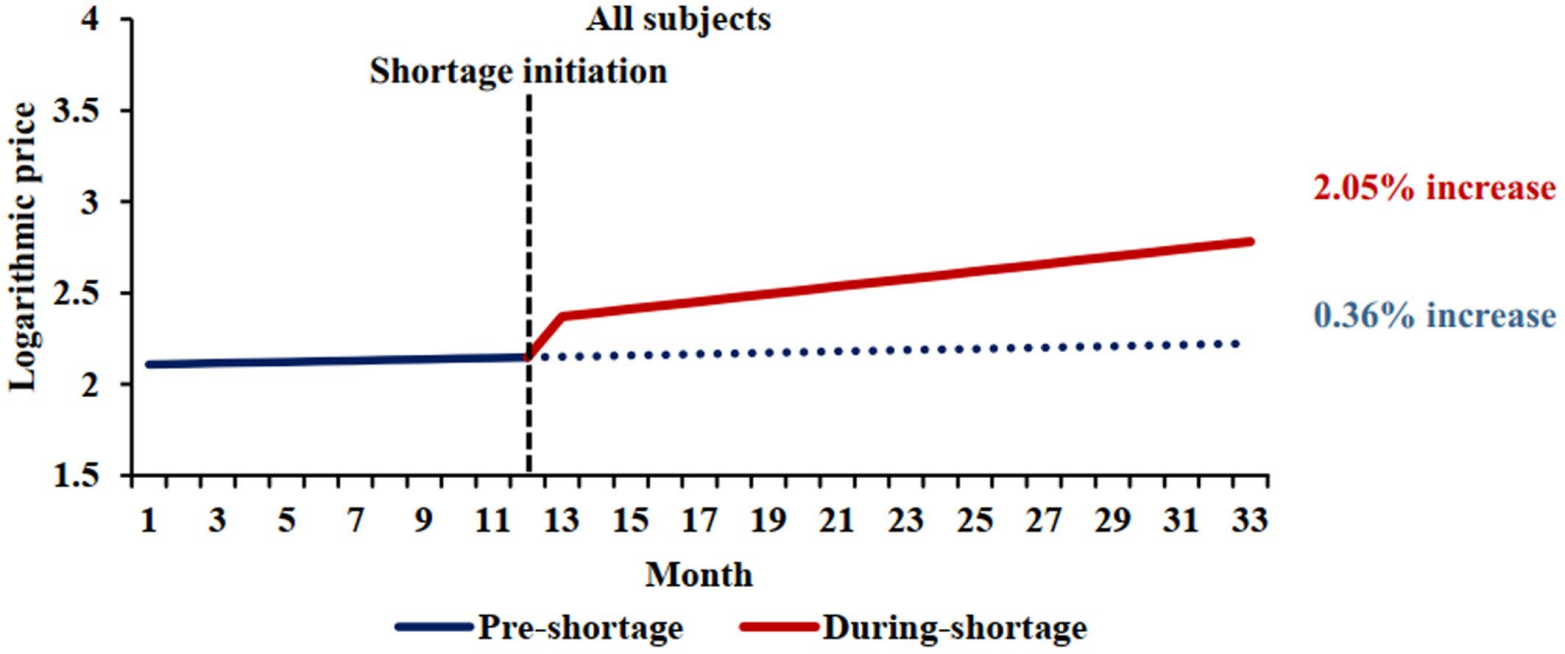
Comparison of pre-shortage and shortage logarithmic price trends. The blue solid line represents the price trend of shortage cohort before shortages, and the red solid line represents the price trend of shortage cohort during shortages. The shortage initiation times was March 2020, which was median shortage initiation month. The blue dotted line shows the price trend of shortage cohort when no shortages occured.

This analysis included 813 shortage markets (364 markets were excluded due to lack of price data in either the pre-shortage or the shortage periods). The mean shortage duration was 5 months (interquartile range: 3, 7). The observed slopes were 0.0036 and 0.0205 during the pre-shortage and shortage periods, respectively (difference = 0.0169, 95%CI 0.0047 to 0.0292; *p* = 0.007) (Table 2 and Figure 1). This difference was statistically significant and corresponded to an annual price increase of 4.4 and 27.6% for the pre-shortage and active shortage periods (difference in annual price = 23.2, 95% CI: 5.8 to 44.3%).

### 3.3. Analysis 3: comparison of shortage and post-shortage periods

**Figure 2:**
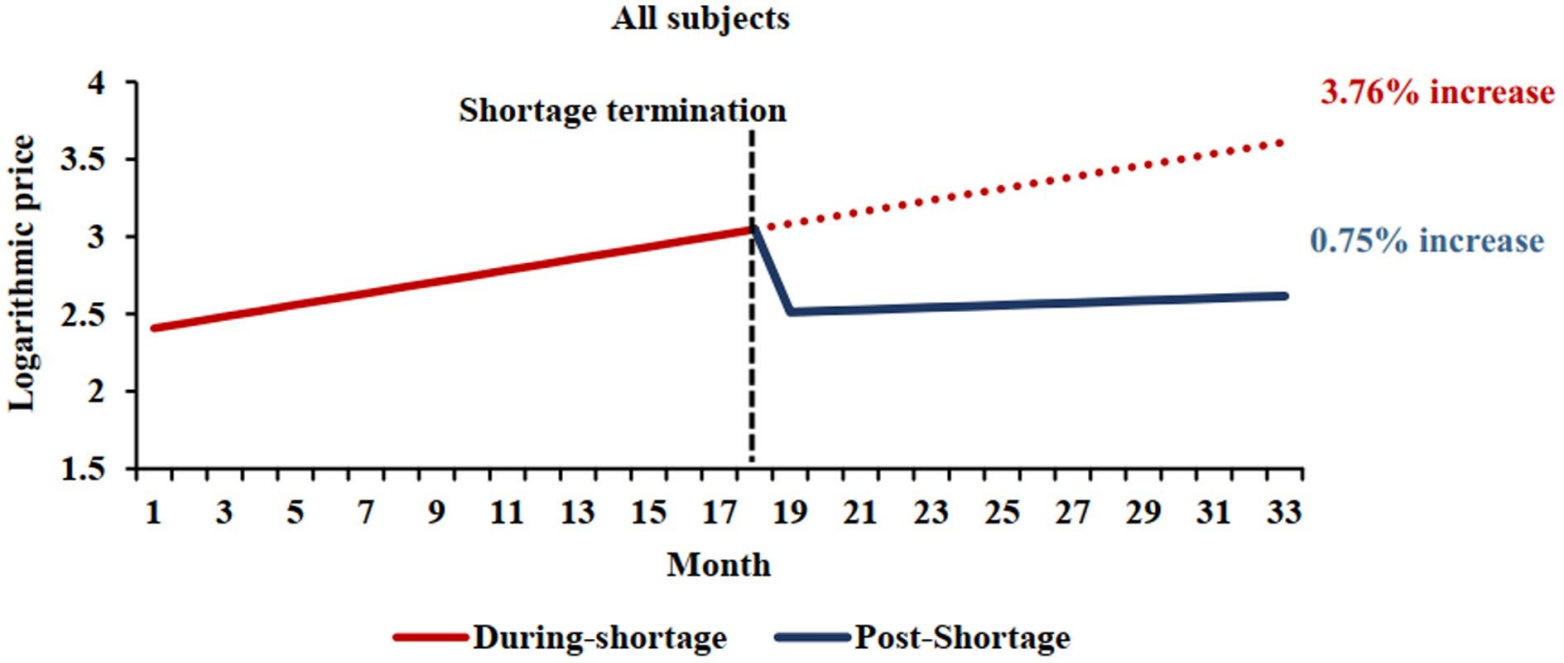
Comparison of shortage and post-shortage logarithmic price trends. The red solid line represents the price trend of shortage cohort during shortage periods, and the blue solid line represents the price trend of shortage cohort during post-shortage periods. The shortage termination times was September 2020, which was median ending month of shortage periods. The red dotted line shows the price trend of shortage cohort if shortages persist.

This analysis included 896 markets (281 markets were excluded due to a lack of data for the shortage and/or post-shortage periods). We observed a slope of 0.0376 during a shortage, whereas post-shortage a slope of 0.0075 was noted (difference = −0.0301, 95%CI −0.0403 to −0.0200; *p* < 0.001) (Table 2 and Figure 2). Consequently, the price of the studied markets increased by 55.73 and 9.38% annually in the shortage and post-shortage periods, respectively (difference in annual price = −46.3, 95% CI: −53.7% to −35.6%).

Notably, the estimated pre-shortage price growth rates for analysis 1 and 2, and the shortage price growth rates for analysis 2 and 3 differ from one another. This is primarily due to differences in the causal contrasts of interest for each model as well as the data inputs. We evaluated the validity of the assumptions of models by observing residual plots, finding no serious violations which may affect our results (Supplementary Appendix 5).

### 3.4. Sensitivity, subgroup, and secondary analyses

Sensitivity analyses were conducted across alternative scenarios where we included the following two, three, four, or all months after the resolution of the drug shortage as the shortage period. Differences across these scenarios’ results were immaterial (Supplementary Appendixes 6, 7).

We performed two subgroup analyses of Analysis 2 and Analysis 3, respectively. Firstly, based on whether the market was sole-supplied or not, we found that there was no statistically significant difference in increases of two slopes between the two subgroups when shortage occurred (*p* = 0.08); whereas, compared with sole-supplied markets, the price growth rate of markets supplied by more than one manufacture declined more after the shortage resolution (*p* = 0.009). Secondly, based on the severity of the shortage, no statistically significant difference was observed in increases of two slopes between the two subgroups when shortage occurred (*p* = 0.96); whereas, the shortage resolution had a greater impact on prices of markets whose delivery rate was below 24.15% (*p* = 0.04) (Supplementary Appendix 3).

In the analysis where we compared the price growth rate during the whole period, we found that the price growth of the non-shortage and low-priced subgroup (1.99%) was significantly higher than that of the non-shortage and high-priced subgroup (−3.31%), and this rule applied also to the shortage cohort. In the two low-priced subgroups, the price growth of shortage subgroup (10.0%) was significantly higher than that of non-shortage subgroup (1.99%); whereas, there was no significant difference between the two high-priced subgroups (Supplementary Appendix 8).

## 4. Discussion

Our study found that the prices of shortage markets increased during their pre-shortage periods, and these increases were not significantly lower than that observed in the non-shortage cohort. Shortages resulted in significant price increases of study markets, especially the low-priced markets, while the shortage resolution slowed the growth. Compared with that of markets supplied by one manufacturer, the growth rates of markets supplied by more than one manufacturer declined more after the shortage resolution. And the shortage resolution had a greater impact on prices of markets with severer shortage.

Our finding that the price changes were not significantly different between the shortage cohort in the pre-shortage period and the non-shortage cohort is likely explained due to the way we defined drug shortages (i.e., 40% delivery rate). To verify it, we compared model results across four scenarios which took the delivery rate less than 30, 20, 10% or 0%, respectively, as the indicator of shortage. We found that only when delivery rate equaled to zero (no supply), the difference in the price changes between the non-shortage cohort and the shortage cohort in the pre-shortage period was significant. Even for those drugs with zero delivery rate, the prices were increasing before their shortages. This result was different with previous evidence from USA, which showed that the price increased in the non-shortage cohort and decreased in the shortage cohort before the shortage (40). It also contradicts prior data which has identified low drug prices as an important factor for drug shortages in China (22–24).

In recent years and with the main objective of ensuring continued access to medications, the central government has implemented several policies to address low drug prices in an effort to combat drug shortages (25, 27, 28). Accordingly, with the resulting rise in drug prices, the primary etiological factor for drug shortages has shifted from low price to other causes, such as unavailability of active pharmaceutical ingredients (APIs) (51–53). Prior literature has shown that the unavailability of API is driven by shortages of raw materials and the implementation of new (and more rigorous) standards set by China Pharmacopoeia requiring manufacturers to re-certify their APIs’ (52, 54). To address this issue, we suggest that the government consider searching alternative APIs sources from other regions and increasing imports. For the latter problem, in addition to providing economic incentive to encourage manufacturers to update new facility for drugs with real low price, Chinese National Medical Products Administration should also expedite the compliance inspection to help with the API certification process.

Regarding the impact of drug shortages on prices, prices increased as expected during the shortage periods due to the increased bargaining power of remaining suppliers (55). While the growth rates in the post-shortage periods declined, drug prices continued to increase steadily following the resolution of the drug shortage. The results conformed to the law of supply–demand: when demand was gradually satisfied, price decelerated and returned to their pre-shortage trend. The results differed from a previous study in USA, which showed that the growth rate remained higher after the shortage resolution (40). This discrepancy in findings can be attributed to differences in drug pricing regulations. For instance, in the United States, pharmaceutical companies set prescription drug prices, which are largely unregulated by the government; However, in China, abnormal price changes are monitored and restricted by the NHSA (56).

Subgroup analysis showed that there was no significant difference in increases of the price growth rate between the sole-supplied subgroup and the subgroup with more than one manufacturer when a shortage occurred, but the price growth rate of markets supplied by more than one manufacturer dropped much more after the shortage resolution. In a perfectly competitive market, the price is determined by the market, and the manufacturers have no marginal profit and passively accept the price; whereas, under an imperfect competitive market, manufacturers determine the price based on their own profit maximization, which is higher than in a perfectly competitive market. Theoretically, compared with that of markets supplied more than one manufacturer, the growth rate of sole-supplied markets should rise faster when a shortage occurred and be more persistent after the shortage resolution. However, the results showed no significant difference in price growth between two subgroups during a shortage. A possible reason for this is that prices of sole-supplied markets were higher to begin with, and thus, a relatively smaller relative increase in price in this instance would correspond to higher absolute increase in price. Governments usually strive to ensure a perfect competitive market for medicines to lower the shortage’s impact on price. The U.S. Food and Drug Administration expedites review of generic drug applications in markets served by three or fewer manufacturers (41). At present, CNMPA only provides one green channel of expediting registration and approval for six chemical entities in the National Shortage Drug List (57). In the future, we can learn from the FDA’s practice, not only preventing shortages and forming a perfect competitive market, but also stabilizing drug prices and reducing the economic burden of patients.

Subgroup analysis also showed that there were no significant difference rates of increases in drug prices between subgroups with different levels of shortage severities during the shortage period. However, after the resolution of the shortage, the price growth in markets with delivery rates below 24.15% declined more significantly. Theoretically, when the supply could not meet the demand, the greater the gap, the more the price increase. However, our research showed that was not the case. One possible reason was that the rule of “less than 40% delivery rate” already represented serious shortages in provincial level, so the increase of the price growth rate of the shortage markets screened by this rule was immaterial. Another reason was the price monitoring implemented by the NHSA. When the manufacturers raised the price, they would set it within the so-called reasonable price range. Otherwise, they had to explain the reasons to NHSA or be warned and punished. Moreover, the data showed that the subgroup with the severer shortage had a longer during-shortage period, resulting in a larger cumulative increase of price growth rate, which explained that its price growth rate dropped more after the shortage resolution.

By comparing the price growth of non-shortage and shortage cohort during the whole period, we found that the price growth of shortage and low-priced subgroup was significantly higher than that of non-shortage and low-priced subgroup. This result re-confirmed that drug shortages lead to a significant price increase. However, there was no significant difference between the price growth rates of two high-priced subgroups, indicating that shortages had a greater impact on prices of low-priced medicines than high-priced ones.

Regarding limitations, we considered the delivery rate as the shortage indicator due to the unavailability of the drug shortage data. The delivery rate calculated by demand and supply quantities can largely reflect the supply situation of drugs in a certain province per month, but it was not equivalent to shortage. More information was needed to define the shortage of a drug, such as the stock and off-take potential. Secondly, we only considered 57 chemical entities including 163 drugs in the study, and these entities were in unstable supply at certain regions of China. More drugs need to be included to generate a more robust result. Moreover, although nearly 92% of the non-shortage markets had an average delivery rate of more than 80%, it’s better to include drugs fully supplied in the non-shortage cohort. Thirdly, we only examined the longest-lasting shortage for each market during the study period. There were a few markets had several shortages; disregard of these shorter shortages would underestimate the impacts of the shortage or shortage resolution on prices. Lastly, the study only included 33 months, a longer period would be better to generate a robust result.

## 5. Conclusion

The prices of shortage drugs were increasing before shortage and had no significant different from non-shortage drugs. However, the shortages were linked to significant price increases of the affected medicines, especially the low-priced markets. Moreover, the shortage of drugs with different number of manufacturers had different impacts on their prices. We suggest the government paying more attention to other drivers of shortage, not just the low price, to prevent or address the drug shortages. In addition, for sole-supplied drugs, the expedited review of applications from other manufacturers should be considered to form a perfect competitive market.

## Data availability statement

The datasets presented in this article are not readily available because they contain confidential information. Requests to access the datasets should be directed to CY, yangcj@xjtu.edu.cn.

## Author contributions

SH and CY: concept and design. JinZ and MP: acquisition of data. JieZ and CX: analysis and interpretation of data. SH: drafting of the manuscript. SH, CD, CY, and YF: critical revision of the manuscript for important intellectual content. CY: obtaining funding. JL: administrative, technical, or logistic support. All authors contributed to the article and approved the submitted version.
