# Supplementary material for: The impact of drug shortages on drug prices: evidence from China
Source: Front Public Health. 2023 Nov 8;11:1185356. doi: 10.3389/fpubh.2023.1185356 (PMC10663221; doi:10.3389/fpubh.2023.1185356)
Supplement: Supplementary file 1 [file Table_1.DOCX]

Supplementary Material

The impact of drug shortages on drug prices:
evidence from China

Shuchen Hu, Jinwei Zhang, Jianwei Li, Jieqiong Zhang, Mengyuan Pan, Cheng Xiang, Chintan V. Dave, Caijun Yang*, Yu Fang*

*** Correspondence:**Caijun Yang: [yangcj@xjtu.edu.cn](mailto:yangcj@xjtu.edu.cn)
Yu Fang: [yufang@mail.xjtu.edu.cn](mailto:yufang@mail.xjtu.edu.cn)

# Appendix 1. 57 chemical entities in the study cohort

Table 1. 57 chemical entities in the study cohort

| **No.** | **Name** | **Dosage forms** | **Strengths** |
| --- | --- | --- | --- |
| 1 | Acetamide | Solution for Injection | 5ml:2.5g |
| 2 | Adernocorticotropime | Sterile Powder for Injection | 25IU |
| 3 | Allopurinol | Sustained-release Capsules | 0.25g |
|  |  | Tablets | 0.1g |
| 4 | Amiodarone | Solution for Injection | 2ml:150mg |
|  |  |  | 3ml:0.15g |
| 5 | Arginine | Solution for Injection | 20ml:5g |
| 6 | Atropine | Solution for Injection | 1ml:0.5mg |
|  |  |  | 1ml:1mg |
|  |  |  | 1ml:5mg |
|  |  |  | 2ml:10mg |
|  |  |  | 2ml:1mg |
| 7 | Benzathine Benzylpenicillin | Sterile Powder for Injection | 1200000IU |
| 8 | Bleomycin | Sterile Powder for Injection | 15000IU |
| 9 | Bleomycin A5 | Sterile Powder for Injection | 8mg |
| 10 | Calcium Gluconate | Solution for Injection | 100ml:1g |
|  |  |  | 10ml:1g |
| 11 | Clofazimine | Soft Capsules | 50mg |
| 12 | Cyclophosphamide | Sterile Powder for Injection | 0.2g |
|  |  |  | 0.5g |
|  |  |  | 1g |
| 13 | Cytarabine | Sterile Powder for Injection | 0.1g |
|  |  |  | 0.3g |
|  |  |  | 0.5g |
|  |  |  | 50mg |
| 14 | Deslanoside | Solution for Injection | 2ml:0.4mg |
| 15 | Diazepam | Solution for Injection | 2ml:10mg |
| 16 | Digoxin | Oral Solution | 30ml:1.5mg |
| 17 | Dobutamine | Solution for Injection | 2ml:20mg |
| 18 | Dopamine | Solution for Injection | 2ml:20mg |
|  |  | Sterile Powder for Injection | 10mg |
|  |  |  | 20mg |
| 19 | Epinephrine | Solution for Injection | 1ml:1mg |
| 20 | Ethacriding | Solution for Injection | 2ml:50mg |
| 21 | Etoposide | Solution for Injection | 2ml:40mg |
|  |  |  | 5ml:100mg |
| 22 | Furosemide | Solution for Injection | 2ml:20mg |
|  |  | Sterile Powder for Injection | 20mg |
|  |  |  | 40mg |
| 23 | Hydrocortisone | Solution for Injection | 20ml:100mg |
|  |  |  | 2ml:10mg |
|  |  |  | 5ml:25mg |
|  |  | Sterile Powder for Injection | 0.1g |
|  |  |  | 50mg |
| 24 | Isoprenaline | Solution for Injection | 2ml:1mg |
| 25 | Lobeline | Solution for Injection | 1ml:3mg |
| 26 | Magnesium sulfate | Solution for Injection | 10ml:1g |
|  |  |  | 10ml:2.5g |
| 27 | Mannitol | Solution for Injection | 100ml:20g |
|  |  |  | 2000m1:100g |
|  |  |  | 250ml:50g |
|  |  |  | 3000ml:150g |
| 28 | Mercaptopurine | Tablets | 50mg |
| 29 | Methotrexate | Solution for Injection | 10ml:1000mg |
|  |  |  | 20ml:500mg |
|  |  | Sterile Powder for Injection | 0.1g |
|  |  |  | 1g |
|  |  |  | 50mg |
|  |  |  | 5mg |
| 30 | Methylthioninium Chloride | Solution for Injection | 2ml:20mg |
| 31 | Mitomycin | Sterile Powder for Injection | 10mg |
| 32 | Mitoxantrone | Sterile Powder for Injection | 5mg |
| 33 | Naloxone | Solution for Injection | 10ml:4mg |
|  |  |  | 1ml:0.4mg |
|  |  |  | 1ml:1mg |
|  |  |  | 2ml:2mg |
|  |  | Sterile Powder for Injection | 0.4mg |
|  |  |  | 0.8mg |
|  |  |  | 1.2mg |
|  |  |  | 1mg |
|  |  |  | 2mg |
|  |  |  | 4mg |
| 34 | Neostigmine Methylsulfate | Solution for Injection | 1ml:0.5mg |
|  |  |  | 2ml:1mg |
|  |  | Sterile Powder for Injection | 1mg |
| 35 | Nikethamide | Solution for Injection | 1.5ml:0.375g |
| 36 | Nitroglycerin | Solution for Injection | 1ml:5mg |
|  |  | Tablets | 0.5mg |
|  |  |  | 0.6mg |
| 37 | Norepinephrine | Solution for Injection | 1ml:2mg |
| 38 | Oxytocin | Solution for Injection | 0.5ml:2.5IU |
|  |  |  | 1ml:10IU |
|  |  |  | 1ml:5IU |
|  |  | Sterile Powder for Injection | 10IU |
| 39 | Penicillamine | Tablets | 0.125g |
| 40 | Phenobarbital | Solution for Injection | 1ml:0.1g |
|  |  | Sterile Powder for Injection | 0.1g |
| 41 | Phentolamine | Solution for Injection | 1ml:10mg |
|  |  | Sterile Powder for Injection | 10mg |
| 42 | Posterior Pituitary | Solution for Injection | 1ml:6IU |
|  |  |  | 2ml:6IU |
| 43 | Pralidxime Chloride | Solution for Injection | 2ml:0.5g |
| 44 | Propatenone | Solution for Injection | 10ml:35mg |
|  |  |  | 20ml:70mg |
| 45 | Protamine | Solution for Injection | 5ml:50mg |
| 46 | Pyridostigmine Bromide | Tablets | 60mg |
| 47 | Snake Antivenins | Solution for Injection | 10000IU |
|  |  |  | 1000IU |
|  |  |  | 2000IU |
|  |  |  | 6000IU |
| 48 | Sodium Dimercaptopropane-sulfonate | Solution for Injection | 2ml:0.125g |
| 49 | Sodium Nitroprusside | Sterile Powder for Injection | 50mg |
| 50 | Sodium Thiosulfate | Sterile Powder for Injection | 0.64g |
| 51 | Thiamazole | Tablets | 10mg |
|  |  |  | 5mg |
| 52 | Thrombin | Lyophilizing Powder | 1000IU |
|  |  |  | 2000IU |
|  |  |  | 200IU |
|  |  |  | 5000IU |
|  |  |  | 500IU |
| 53 | Tretinoin | Tablets | 10mg |
|  |  |  | 20mg |
| 54 | Urokinase | Sterile Powder for Injection | 100000IU |
|  |  |  | 10000IU |
|  |  |  | 250000IU |
| 55 | Verpamil | Solution for Injection | 2ml:5mg |
|  |  | Sterile Powder for Injection | 10mg |
|  |  |  | 5mg |
| 56 | Vineristine | Sterile Powder for Injection | 1mg |
| 57 | Vitamin K1 | Solution for Injection | 1ml:10mg |

# Model equations

Table 2. Model equations

| **The First Section: Comparison of shortage and non-shortage cohorts** |
| --- |
| E(Y_ij_\|b_i_) = β_0_ + β_1_t_ij_ + β_2_t_ij_*shortage_category_ij_ + β_3_drug_category_ij_ + β_4_manufacturers_ij_ + e_ij_ + b_0i_ + b_1itij_  **Variable**  **Y_ij_** is the longarithmic drug price for a given market i at time j.  **t_ij_** is time modelled as a dummy variable indicating the study period. It takes a positive value ranging from 1 to 33 and indicates the month, starting from the April 2019.  **shortage_category_ij_** is a dummy variable indicating whether drug i is in the shortage cohort. It thus takes the value 0 in the non-shortage cohort and the value 1 for the shortage cohort.  **drug_category_ij_** is a variable indicating medication therapeutic class.  **manufacturers_ij_** is a variable indicating the number of suppliers.  **e_ij_** indicates the random error term.  **b_0i_** indicates random intercepts for each market i.  **b_1itij_** indicates random slopes of each market i for the effect of t_ij_.  **Coefficient**  **β_0_** is the overall model intercept.  **β_1-4_** are the fixed-effect coefficients.  We hypothesize that there is no difference in slopes between two categories, i.e H_0_ : β_2_ =0. |
| **The Second Section: Comparison of pre-shortage and shortage price trends** |
| E(Y_ij_\|b_i_) = β_0_ + β_1_t_ij_ + β_2_(t_ij_)_+_ + β_3_drug_category_ij_ + β_4_manufacturers_ij_ + e_ij_ + b_0i_ + b_1itij_  Compared with the first Section, there is no change except the encoding of t_ij_ and (t_ij_)_+_.  In this equation, t_ij_ (time) is coded as time since shortage initiation. It thus takes the value 0 in the first month of shortage and positive values thereafter, and accordingly, negative values for the period before the shortage. For this equation, (t_ij_)_+_= t_ij_ if t_ij_>0 and (t_ij_)_+_=0 if t_ij_≤0.  Our goal was to assess whether drug slopes differ before and after shortage, i.e. H_0_ : β_2_=0. |
| **The Third Section: Comparison of shortage and post-shortage price trends** |
| E(Y_ij_\|b_i_) = β_0_ + β_1_t_ij_ + β_2_(t_ij_)_+_ +β_3_drug_category_ij_ + β_4_manufacturers_ij_ + e_ij_ + b_0i_ + b_1itij_  Compared with the first Section, there is no change except the encoding of t_ij_ and (t_ij_)_+_.  In this equation, t_ij_ (time) is coded as time since shortage termination. It thus takes the value 0 in the first post-shortage month and positive values thereafter, and accordingly, negative values for the shortage period. For this equation, (t_ij_)_+_= t_ij_ if t_ij_>0 and (t_ij_)_+_=0 if t_ij_≤0.  Our goal was to assess whether drug slopes differ before and after shortage, i.e. H_0_ : β_2_=0. |
| **Subgroup analysis of the second and the third section** |
| E(Y_ij_\|b_i_) = β_0_ + β_1_t_ij_ + β_2_(t_ij_)_+_ +β_3_t_ij_*manufacturer_category_ij_ + β_4_(t_ij_)_+_*manufacturer_category_ij_ + β_5_ drug_category_ij_ + β_6_manufacturers_ij_ + e_ij_ + b_0i_ + b_1itij_  Compared with the second and the third section, we added a variable manufacturer_category_ij_. In this equation, t_ij_ (time) and (t_ij_)_+_ are coded in the same way as before; manufacturer_category_ij_ is coded as 0 for single-supplied markets and 1 for markets with more than one manufacturer.  Our goal was to assess whether the difference of drug slopes before and after shortage differ, i.e. H_0_ : β_4_=0. |
| E(Y_ij_\|b_i_) = β_0_ + β_1_t_ij_ + β_2_(t_ij_)_+_ +β_3_t_ij_* delivery rate_ij_ + β_4_(t_ij_)_+_*delivery rate_ij_ + β_5_drug_category_ij_ + β_6_manufacturers_ij_ + e_ij_ + b_0i_ + b_1itij_  Compared with the second and the third section, we added a variable delivery rate_ij_. In this equation, t_ij_ (time) and (t_ij_)_+_ are coded in the same way as before; delivery rate_ij_ is coded as 0 for the markets whose delivery rate was from 24.15% to 40% and 1 for the markets whose delivery rate was lower than 24.15%.  Our goal was to assess whether the difference of drug slopes before and after shortage differ, i.e. H_0_ : β_4_=0. |

# Subgroup analyses

**Methods**

We performed two subgroup analyses of the second and third sections based on the number of manufacturers and the severity of shortage, respectively. Firstly, we divided markets into 2 subgroups according to whether they were supplied by a single manufacturer. In the subgroup analysis of the second section, we assumed that each subgroup had a two-piece linear growth curve with a knot at the time of shortage initiation. The growth curve for each subgroup can thus be described with an intercept (initial price) and two slopes: one slope expressing changes before a shortage and another slope expressing changes during a shortage period. We examined whether there was a difference in difference of two slopes between subgroups. The same approach was employed in the subgroup analysis of the third section. Secondly, we divided markets into 2 subgroups according to whether the delivery rate of first month in shortage period was lower than the median value (24.15%). The difference-in-difference estimation between slopes was the same as in the first subgroup analysis.

**Results**

(1) First subgroup analyses based on the number of manufactures

Based on whether the market was single-supplied or not, we performed a subgroup analysis for the second and third sections, respectively. Prices of single-supplied markets increased by 0.52% and 1.32% per month before and after the shortage initiation, respectively (difference = 0.81%, 95%CI -0.84 to 2.45; p = .34); whereas, for markets supplied by more than one manufacturer, prices increased by 0.10% and 2.91%, respectively (difference = 2.81%, 95%CI 1.21 to 4.44; p < .001) (Table 3, Figure 1). Mixed models showed that the shortage had a statistically significant impact on prices of markets supplied by more than one manufacturer, while the other subgroup was not; There was no statistically significant difference in increases of two slopes between the two subgroups (p = .08).

Prices of single-supplied markets increased by 2.81% and 0.68% per month during a shortage and in post-shortage periods, respectively (difference =-2.13%, 95%CI -3.48 to -0.79; p = .002); whereas, for markets supplied by more than one manufacturer, prices increased by 5.11% and 0.79%, respectively (difference = -4.32%, 95%CI -5.90 to -2.75; p < .001) (Table 3, Figure 2). Mixed models also showed that the shortage resolution had a greater impact on prices of markets supplied by more than one manufacturer (p = .009).

(2) Second subgroup analyses based on the severity of shortage

Based on the delivery rate of the first month in shortage period, we performed the other subgroup analysis for the second and third sections, again. Prices of markets whose delivery rate was below 24.15% (this was the median delivery rate for all the shortage markets) increased by 0.23% and 2.01% per month before and after the shortage initiation, respectively (difference = 1.77%, 95%CI -0.13 to 3.67; p = .07); whereas, for markets whose delivery rate was during the range of 24.15% to 40%, prices increased by 0.46% and 2.10% (difference = 1.63%, 95%CI 0.07 to 3.21, p = .04), respectively (Table 3, Figure 3). Mixed models showed that the shortage had a statistically significant impact on prices of both subgroups. There was no statistically significant difference in increases of two slopes between the two subgroups (p = .96).

Prices of markets whose delivery rate was below 24.15% increased by 4.64% and 0.63% per month during a shortage and in post-shortage periods, respectively (difference = -4.01%, 95%CI -5.52 to -2.50; p < .001); whereas, for markets whose delivery rate was during the range of 24.15% to 40.00%, prices increased by 2.89% and 0.85%, respectively (difference = -2.03%, 95%CI -3.39 to -0.68; p = .003) (Table 3, Figure 4). Mixed models showed that the shortage resolution had a greater impact on prices of markets whose delivery rate was below 24.15% (p = .04).

**0.36% increase**

**2.05% increase**

**Shortage initiation**

**Markets supplied by ＞ 1 manufacturer**

**Shortage initiation**

**Markets supplied by 1 manufacturer**

**1.32% increase**

**0.52% increase**

**0.10% decrease**

**2.91% increase**

**Shortage initiation**

**Figure 1.** Subgroup Analysis 1: Comparison of pre-shortage and shortage logarithmic price trends

**0.75% increase**

**3.76% increase**

**0.79% increase**

**5.11% increase**

**2.81% increase**

**0.68% increase**

**Figure 2.** Subgroup Analysis 1: Comparison of shortage and post-shortage logarithmic price trends

**0.36% increase**

**2.05% increase**

**Markets whose delivery rate is from 0.00% to 24.15%**

**Shortage initiation**

**2.01% increase**

**0.23% increase**

**Markets whose delivery rate is from 24.15% to 40.00%**

**Shortage initiation**

**0.46% increase**

**2.10% increase**

**Shortage initiation**

**Figure 3.** Subgroup Analysis 2: Comparison of pre-shortage and shortage logarithmic price trends

**0.75% increase**

**3.76% increase**

**Markets whose delivery rate is from 0.00% to 24.15%**

**0.63% increase**

**4.64% increase**

**0.95% increase**

**2.89% increase**

**Figure 4.** Subgroup Analysis 2: Comparison of shortage and post-shortage logarithmic price trends

**Table 3.** Logarithmic price trends of three periods between subgroups

|  | **Subgroup Analysis 1** | | | | **Subgroup Analysis 2** | | | |
| --- | --- | --- | --- | --- | --- | --- | --- | --- |
|  | **a single manufacturer** | | **more than one manufacturer** | | **0-24%** | | **24%-40%** | |
|  | **Value(95%CI)** | **P Value** | **Value(95%CI)** | **P Value** | **Value(95%CI)** | **P Value** | **Value(95%CI)** | **P Value** |
| **Comparison of Pre-Shortage and Shortage Periods** |  |  |  |  |  |  |  |  |
| Pre-shortage | 0.52%(0.08%,0.96%) | .02 | 0.10%(-0.37%,0.57%) | .67 | 0.23%(-0.30%,0.77%) | .39 | 0.46%(0.08%,0.85%) | .02 |
| During shortage | 1.32%(-0.77%,3.41%) |  | 2.91%(0.83%,5.01%) |  | 2.01%(-0.43%,4.44%) |  | 2.10%(0.14%,4.06%) |  |
| Difference | 0.81%(-0.84%,2.45%) | .34 | 2.81%(1.21%,4.44%) | < .001 | 1.77%(-0.13%,3.67%) | .07 | 1.63%(0.07%,3.21%) | .04 |
| Difference in difference in slopes between the 2 subgroups | .08 | | | | .96 | | | |
| **Comparison of Shortage and Post-Shortage Periods** |  |  |  |  |  |  |  |  |
| During shortage | 2.81%(1.71%,3.91%) | < .001 | 5.11%(3.76%,6.47%) | < .001 | 4.64%(3.38%,5.90%) | < .001 | 2.89%(1.74%,4.04%) | < .001 |
| Post-shortage | 0.68%(-1.76%,3.12%) |  | 0.79%(-2.15%,3.72%) |  | 0.63%(-2.15%,3.40%) |  | 0.85%(-1.65%,3.36%) |  |
| Difference | -2.13%(-3.48%,-0.79%) | .002 | -4.32%(-5.90%,-2.75%) | < .001 | -4.01%(-5.52%,-2.50%) | < .001 | -2.03%(-3.39%,-0.68%) | .003 |
| Difference in difference in slopes between the 2 subgroups | .009 | | | | .04 | | | |

# The monthly proportion of shortage markets, April 2019 – December 2021

# Figure 5. The monthly proportion of shortage markets, April 2019 – December 2021.

# Validity check of model assumptions


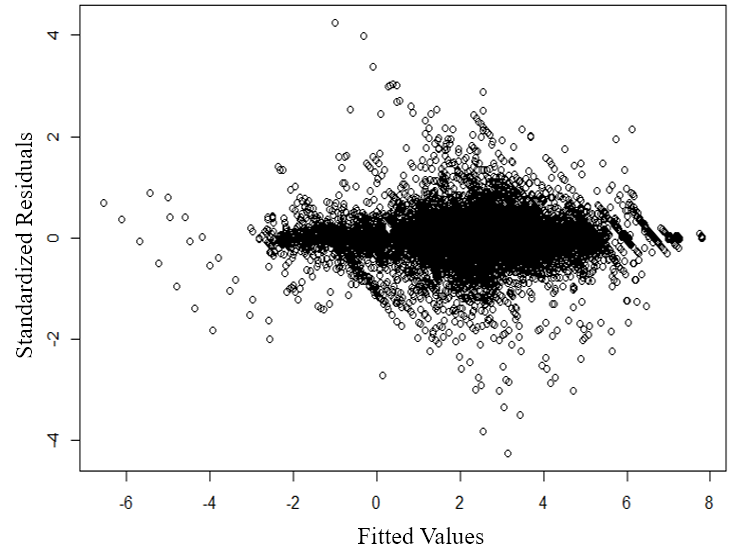


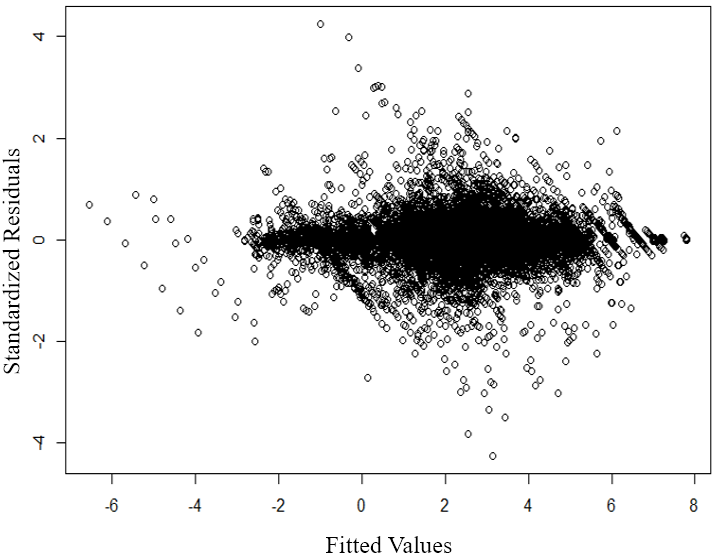


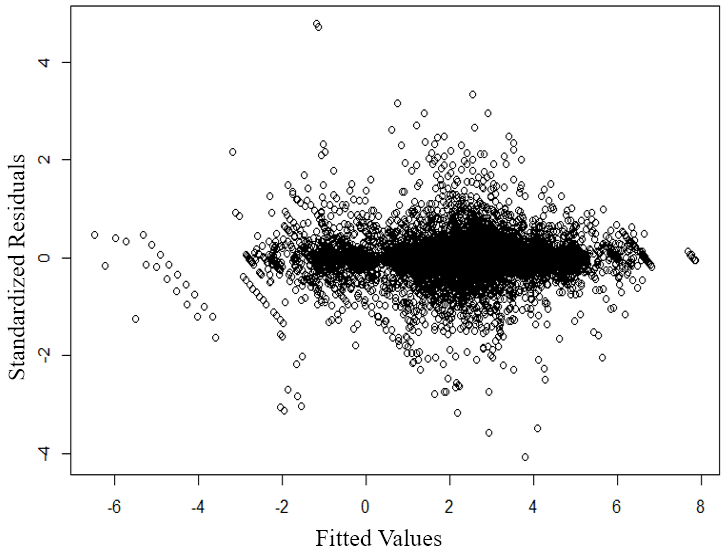


**Figure 6.** Validity check of model assumptions

# The results of the first scenario

# Considering shortage months and the next two months as shortage periods (the price in shortage periods were impacted by the shortage) if shortage lasted at least 2 months.

# Table 4. Logarithmic price trends in pre-shortage, shortage and post-shortage periods

| **Main Analysis** | **Value(95%CI)** | | **P Value** | |
| --- | --- | --- | --- | --- |
| **Comparison of Price Growth in Non-Shortage Periods** |  | |  | |
| **Slope** |  | |  | |
| Non-shortage cohort | | 0.92%(0.67%,1.17%) | | < .001 |
| Shortage cohort | | 0.65%(0.01%,1.30%) | |  |
| Difference | | -0.26%(-0.66%,0.13%) | | .19 |
| **Other fixed effects** | |  | |  |
| Medication class | | 6.89%(4.62%,9.16%) | | < .001 |
| Manufacturers | | -14.03%(-17.61%,-10.46%) | | < .001 |
| **Comparison of Pre-Shortage and Shortage Periods** | |  | |  |
| **Slope** | |  | |  |
| Pre-shortage | | 0.32%(0.00%,0.64%) | | .05 |
| During shortage | | 1.98%(0.20%,3.75%) | |  |
| Difference | | 1.66%(0.21%,3.11%) | | .03 |
| **Other fixed effects** | |  | |  |
| Medication class | | 11.16%(7.62%,14.70%) | | < .001 |
| Manufacturers | | -13.13%(-20.77%,-5.50%) | | < .001 |
| **Comparison of Shortage and** **Post-Shortage Periods** | |  | |  |
| **Slope** | |  | |  |
| During shortage | | 4.17%(3.17%,5.17%) | | < .001 |
| Post-shortage | | 0.98%(-1.16%,3.13%) | |  |
| Difference | | -3.19%(-4.32%,-2.05%) | | < .001 |
| **Other fixed effects** | |  | |  |
| Medication class | | 10.99%(7.87%,14.11%) | | < .001 |
| Manufacturers | | -18.24%(-24.99%,-11.50%) | | < .001 |

# The results of the fifth scenario

Considering shortage months and all the following months as shortage periods if shortage lasted at least 2 months.

**Table 5.** Logarithmic price trends in pre-shortage, shortage and post-shortage periods

| **Main Analysis** | **Value(95%CI)** | **P Value** |
| --- | --- | --- |
| **Comparison of Price Growth in Non-Shortage Periods** |  |  |
| **Slope** |  |  |
| Non-shortage cohort | 0.92%(0.67%,1.17%) | < .001 |
| Shortage cohort | 0.65%(0.01%,1.30%) |  |
| Difference | -0.26%(-0.66%,0.13%) | .19 |
| **Other fixed effects** |  |  |
| Medication class | 6.89%(4.62%,9.16%) | < .001 |
| Manufacturers | -14.03%(-17.61%,-10.46%) | < .001 |
| **Comparison of Pre-Shortage and Shortage Periods** |  |  |
| **Slope** |  |  |
| Pre-shortage | 0.56%(0.22%,0.90%) | .001 |
| During shortage | 1.33%(0.32%,2.35%) |  |
| Difference | 0.78%(0.10%,1.45%) | .02 |
| **Other fixed effects** |  |  |
| Medication class | 11.26%(7.79%,14.73%) | < .001 |
| Manufacturers | -13.90%(-21.47%,-6.33%) | < .001 |
| **Comparison of Shortage and Post-Shortage Periods** |  |  |
| **Slope** |  |  |
| During shortage | 4.97%(2.91%,7.03%) | < .001 |
| Post-shortage | 0.82%(-3.41%,5.06%) |  |
| Difference | -4.15%(-6.32%,-1.97%) | < .001 |
| **Other fixed effects** |  |  |
| Medication class | 7.13%(2.09%,12.16%) | .006 |
| Manufacturers | -18.23%(-28.38%,-8.08%) | < .001 |

# Comparison of price growth in the whole study period

# Table 6. Comparison of price growth in the whole study period

| **Subgroup** | **The median price growth** | **Adjusted. P value** | | | |
| --- | --- | --- | --- | --- | --- |
|  |  | **Subgroup 1** | **Subgroup 2** | **Subgroup 3** | **Subgroup 4** |
| **Subgroup 1** | 1.99% | —— | —— | —— | —— |
| **Subgroup 2** | -3.31% | < .001 | —— | —— | —— |
| **Subgroup 3** | 10.02% | .013 | < .001 | —— | —— |
| **Subgroup 4** | -3.31% | < .001 | 1.00 | < .001 | —— |

Note: Subgroup 1: Non-shortage and low-priced subgroup; Subgroup 2: Non-shortage and high-priced subgroup; Subgroup 3: Shortage and low-priced subgroup; Subgroup 4: Shortage and high-priced subgroup.
